# Adapting Old Tools for the Reality of the Southern Hemisphere: SNP Panels for Local Parentage Assignment in Small Ruminant Breeds from Brazil

**DOI:** 10.3390/ani16213521

**Published:** 2026-11-07

**Authors:** Camila Souza Rodrigues, Danielle Assis de Faria, Samuel Rezende Paiva, Hymerson Costa Azevedo, Kleibe de Moraes Silva, Olivardo Facó, Danielle Maria Machado Ribeiro Azevêdo, Concepta McManus

**Affiliations:** 1 Faculty of Agronomy and Veterinary Medicine, Central Institute of Sciences, Darcy Ribeiro Campus, University of Brasília, Brasília 70910-900, DF, Brazil; rodriguesscamila@outlook.com (C.S.R.); concepta@unb.br (C.M.); 2 Embrapa Genetic Resources and Biotechnology, Brasília 70770-917, DF, Brazil; samuel.paiva@embrapa.br; 3 Embrapa Coastal Tablelands, Aracaju 49025-040, SE, Brazil; hymerson.azevedo@embrapa.br; 4 Embrapa Goats and Sheep, Sobral 62010-970, CE, Brazil; kleibe.silva@embrapa.br (K.d.M.S.); olivardo.faco@embrapa.br (O.F.); 5 Embrapa Mid-North, Teresina 64008-780, PI, Brazil; danielle.azevedo@embrapa.br

**Keywords:** genetic resources, parentage testing, pedigree, simulation analysis, breed conservation

## Abstract

This study evaluated species-specific 96-SNP panels for parentage assignment in Brazilian sheep and goats using genotypes from 1481 animals, generated with the EMBRAPA Multispecies 65K Illumina Infinium 1 chip. Panels were selected under strict quality control (minor allele frequency ≥ 0.30) and compared with published panels and full species-specific datasets. Evaluation included confirmed trios (84 sheep, 95 goats) and independent simulation sets (357 sheep, 117 goats). Under the P2 parentage-exclusion scenario, in which the second parent’s genotype is unknown, the panels achieved a cumulative probability of exclusion (CPE) of 0.9999 using ≤70 SNPs in sheep and ≤72 in goats. They showed higher heterozygosity and polymorphic information content than random subsets and published reference panels (Holm-adjusted *p* < 0.0001), whereas differences from the full species-specific datasets varied. In empirical trio validation, observed parentage assignment success at the 95% confidence level was 96.43% in sheep and 95.80% in goats, comparable to larger panels. These findings support the potential utility of the panels for parentage assignment. As all markers are present on the multispecies BeadChip, they can be extracted from existing genotyping datasets, potentially reducing additional genotyping requirements and computational burden. Further validation across broader genetic backgrounds and complex pedigree scenarios is recommended before large-scale implementation.

## Simple Summary

DNA-based parentage testing provides an objective approach to verifying pedigree relationships. However, high-density SNP genotyping may be less accessible to small herds, which account for a substantial share of Brazilian production of locally adapted sheep and goats, because of its cost and associated analytical and operational requirements. Using the EMBRAPA Multispecies 65K Illumina Infinium 1 chip, this study selected and evaluated reduced panels of 96 genetic markers for parentage testing in Brazilian sheep and goats. These panels were compared with larger existing marker sets using measures of genetic diversity and empirical parentage assignment in trios with recorded pedigrees. The 96-marker panels retained high genetic informativeness and achieved parentage assignment success rates of 96.43% in sheep and 95.80% in goats, comparable to those obtained with larger marker sets. Because all selected markers are part of the existing Embrapa chip, these panels can be extracted from existing genotyping datasets, potentially reducing the need for additional genotyping and simplifying downstream analyses. These findings support the use of reduced marker panels for parentage testing in Brazilian small ruminants and provide a foundation for developing more accessible, targeted genetic testing tools.

## 1. Introduction

Effective characterization, conservation, and utilization of genetic resources are essential for achieving sustainable sheep and goat production. Accurate pedigree records are critical for predicting estimated breeding values (EBV) and optimizing selection and mating strategies in both commercial breeding and genetic conservation programs [1]. While microsatellites (short tandem repeats, STRs) have historically been widely used for parentage testing in small ruminants [2,3,4,5], single-nucleotide polymorphisms (SNPs) have emerged as the preferred alternative. As highlighted by the International Society for Animal Genetics (ISAG), expanding and benchmarking SNP panels is important for establishing reliable parentage-testing standards, because SNPs offer lower mutation rates, more uniform genomic distribution, and greater interlaboratory reproducibility than STRs [6,7,8].

Exclusion probability remains a primary criterion for evaluating the discriminatory power of SNP panels. In exclusion-based parentage testing, candidate parents may be excluded when their genotypes are incompatible with Mendelian inheritance. Markers with high minor allele frequency (MAF) and high heterozygosity are particularly valuable for maximizing cumulative exclusion power [9]. Because exclusion probability is strongly influenced by allele frequencies, markers with relatively high minor allele frequencies (MAF ≥ 0.30) may be useful when developing panels intended for use across multiple populations [9]. Exclusion-based analyses can also be complemented by likelihood-based approaches, which incorporate allele frequencies, genotyping errors, and pedigree structure to support parentage assignment under complex scenarios [7].

SNP-based parentage testing has reached a high degree of standardization in several livestock species. For instance, the ISAG–ICAR cattle panel includes internationally standardized core and additional SNP sets selected for high exclusion power across diverse *Bos taurus* and *Bos indicus* breeds [10]. In small ruminants, several SNP panels have been proposed for parentage assignment [9,11,12,13,14,15,16,17]. However, while these panels were developed using geographically diverse populations, their empirical evaluations were conducted primarily in North American and European cosmopolitan breeds [9,17]. Thus, locally adapted Brazilian sheep and goats remain comparatively underrepresented in parentage panel benchmarking. This represents a critical knowledge gap, as Brazilian small ruminants have undergone generations of adaptation to distinct local biomes, leading to genetic divergence from North American and European reference breeds that may reduce the informative power of standard markers [18,19,20].

Furthermore, small ruminant production in developing tropical regions poses distinct operational constraints. Production is largely concentrated among smallholder farmers where formal animal identification and pedigree recording are limited [21,22]. Under such circumstances, reliable molecular parentage testing can enable pedigree reconstruction, support herd management, and strengthen genetic conservation programs. However, high-density SNP genotyping is often cost-prohibitive for non-commercial and conservation herds. Therefore, reduced SNP panels that retain high discriminatory power while being tailored to local genetic backgrounds are urgently needed.

Recently, the EMBRAPA Multispecies 65K Illumina Infinium 1 chip, a cost-efficient genotyping platform, was developed, comprising 66,413 SNPs across 27 plant and animal species of relevance to Brazilian agriculture [23,24,25,26]. For *Ovis aries*, the platform contains a curated subset of 2926 ovine-specific SNPs, derived from the commercial OvineSNP50 BeadChip, while for *Capra hircus*, it includes 1492 SNPs from the CaprineSNP50 array. Although a multispecies array requires careful consideration of probe specificity, the locus-specific hybridization chemistry of the Infinium platform minimizes cross-species signal interference. In the present study, analyses were restricted to the ovine and caprine subsets, which were subjected to stringent quality-control filtering. This study aimed to identify and empirically evaluate reduced panels of highly informative SNPs for parentage assignment in Brazilian sheep and goats. The resulting panels were evaluated using cumulative exclusion probabilities and empirical assignment accuracy, and benchmarked against full species-specific datasets and published reference panels. This approach provides a population-informed framework for reducing marker density while retaining high discriminatory power for smallholder, conservation, and commercial breeding applications.

## 2. Materials and Methods

### 2.1. Samples and Genotyping Data

A total of 1481 DNA samples retrieved from the Banco de DNA e Tecidos Animais biobank at Embrapa Genetic Resources and Biotechnology were genotyped using the EMBRAPA Multispecies 65K Illumina Infinium 1 chip (Neogen/GeneSeek, Lincoln, NE, USA, https://www.neogen.com/, accessed on 22 September 2026). This BeadChip comprises 2926 SNPs in sheep (distributed across 26 autosomes) and 1492 SNPs in goats (across 29 autosomes and the X chromosome). Genotype calling was performed using GenomeStudio v2.0 (Illumina Inc., San Diego, CA, USA, https://www.illumina.com/, accessed on 22 September 2026) following standard quality-control procedures, and allele calls were exported in TOP format (A, C, G, and T). Additionally, previously generated genotyping data for these animals from the OvineSNP50 and CaprineSNP50 BeadChips (Illumina, San Diego, CA, USA, https://www.illumina.com/, accessed on 22 September 2026) were used to retrieve genotype information for the 163 and 195 SNPs comprising the sheep and goat reference published panels, respectively. Because this study relied exclusively on existing biobank specimens and involved no live animal handling or primary tissue collection by the authors, formal ethical approval from an institutional animal care and use committee was not required.

The initial sample set used for SNP selection comprised 439 individuals from locally adapted Brazilian sheep breeds: Santa Inês (*n* = 148), Morada Nova (*n* = 44), Crioula (*n* = 227), and Brazilian Somali (*n* = 20). For goats, 197 individuals were included, comprising locally adapted breeds—Azul (*n* = 15), Canindé (*n* = 25), Marota (*n* = 40), Moxotó (*n* = 37) and Repartida (*n* = 24)—and cosmopolitan breeds (Alpine (*n* = 19), Anglo-Nubian (*n* = 12), and Saanen (*n* = 25)).

Marker selection included both locally adapted Brazilian breeds and cosmopolitan dairy breeds (Alpine, Anglo-Nubian, and Saanen). Including cosmopolitan breeds allowed marker selection to account for variation across the goat populations represented in the dataset, while maintaining a focus on the potential application of the panel to locally adapted Brazilian breeds. However, the inclusion of these breeds during marker selection does not establish equivalent panel performance across populations, which must be assessed through empirical validation.

Subsequently, panel performance was evaluated using an independent sample set, consisting of trios (sire, dam, and offspring) with confirmed pedigree records. A total of 84 sheep trios (175 unique individuals, as some sires and dams were shared across offspring) were analyzed across four breeds: Brazilian Somali (*n* = 10 trios), Morada Nova (*n* = 15 trios), Crioula (*n* = 21 trios), and Santa Inês (*n* = 38 trios). For goats, 95 trios were evaluated (representing 196 unique individuals due to repeated parents) across five breeds: Canindé (*n* = 10 trios), Moxotó (*n* = 6 trios), Azul (*n* = 4 trios), Marota (*n* = 5 trios), and Saanen (*n* = 70 trios).

Saanen accounted for 70 of the 95 empirical caprine trios (73.7%). This predominance reflects the commercial importance of Saanen as a dairy breed in Brazil and the greater availability of animals for empirical parentage validation compared with locally adapted Brazilian breeds. These locally adapted breeds are often maintained in relatively small herds, limiting the number of available animals and, consequently, the number of complete parentage trios that can be assembled for empirical evaluation. The inclusion of Saanen therefore allowed the panel to be evaluated in a commercially important breed, while the locally adapted breeds were represented by the trios available for this study. Nevertheless, the unequal breed representation should be considered when interpreting the empirical validation results.

Furthermore, to complement the empirical trio validation, a separate parentage simulation analysis was conducted for each species using an independent sample set comprising animals that were not included in the empirical trio validation. For sheep, 357 individuals were analyzed: Santa Inês (*n* = 225), Morada Nova (*n* = 95), and Crioula (*n* = 37). For goats, 117 animals were analyzed: Azul (*n* = 4), Marota (*n* = 14), Canindé (*n* = 31), Moxotó (*n* = 30), and Saanen (*n* = 38). Individuals were selected to minimize close pedigree relationships. Genotypes for the selected 96-SNP panels, the published reference panels (163 SNPs for sheep and 195 SNPs for goats), and the full species-specific datasets were extracted to enable direct performance comparisons. A summary of the sample sets used at each stage of the study is provided in Supplementary Table S1.

### 2.2. Quality Control and SNP Selection

**Figure 1:**
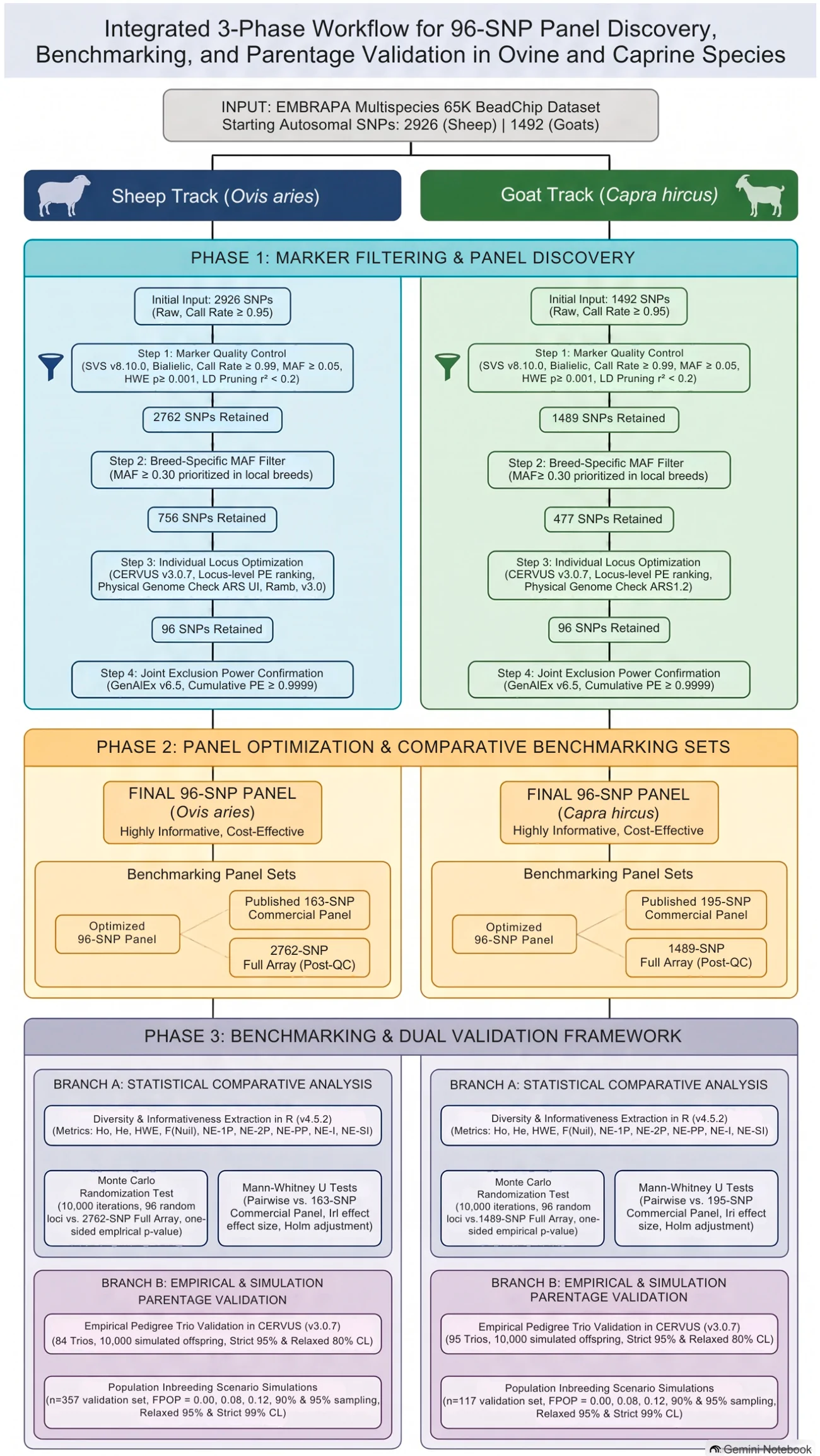
Integrated 3-Phase workflow for 96-SNP panel discovery, comparative statistical benchmarking, and parentage assignment validation in sheep (*Ovis aries*) and goats (*Capra hircus*).

SNP selection followed a four-step procedure (Figure 1), performed separately for each species using the selection sample sets described in Section 2.1 (439 sheep and 197 goats). These animals were not used in the empirical trio assignment or in the simulation analyses (Section 2.4).

Step 1: Quality control. This step was performed using SNP and Variation Suite (SVS) v8.10.0 (Golden Helix, Inc., Bozeman, MT, USA). Samples with a call rate < 0.95 were excluded. Only biallelic SNPs with a call rate ≥ 0.99 and a minor allele frequency (MAF) ≥ 0.05 were retained, and SNPs deviating from Hardy–Weinberg equilibrium (HWE; *p* < 0.001) were excluded. Linkage disequilibrium (LD) pruning was subsequently carried out using a sliding window approach (window size of 50 markers, step size of 5 markers). LD between marker pairs was quantified using the *r*^2^ statistic via the Composite Haplotype Method (CHM), and one SNP from any pair exceeding *r*^2^ > 0.2 was removed. These filters were applied to all breeds within each species, resulting in a refined set of high-quality markers retained for downstream panel selection: 2762 for sheep and 1489 for goats.

Step 2: Identification of candidate SNPs. From these retained markers, candidate SNPs for small parentage panels were selected based on breed-specific allele frequency rankings and genomic distribution. Minor allele frequencies (MAF) were calculated separately for each breed within both species (sheep and goats). The markers were then ranked by MAF in descending order within each breed side-by-side. SNPs with MAF ≥ 0.30 in every breed included in marker selection were prioritized to favor highly informative loci across the sampled populations. This threshold was selected because relatively balanced allele frequencies generally increase the exclusion power of individual SNPs, and similar MAF thresholds have been used in sheep parentage panel development [9]. Requiring the threshold to be met in every breed was a stringent selection criterion intended to prioritize cross-breed informativeness, although it reduced the pool of eligible markers and could exclude loci that were informative in some breeds but less polymorphic in others. To ensure genome-wide coverage, selected SNPs were cross-referenced against their physical genomic positions using the sheep reference genome ARS-UI_Ramb_v3.0 (GCF_016772045.2) and the goat reference genome ARS1.2 (GCA_001704415.2). Where necessary, individual candidate SNPs were replaced with nearby high-MAF markers to fill coverage gaps. This procedure yielded 756 candidate SNPs in sheep and 477 in goats.

Step 3: SNP ranking and panel assembly. The informativeness of each candidate SNP (756 in sheep and 477 in goats) was assessed using CERVUS v3.0.7 [27], including observed and expected heterozygosity (*H_o_* and *H_e_*), polymorphic information content (PIC), probability of exclusion (PE), and non-exclusion probabilities (NE-1P, NE-2P, NE-PP, NE-I and NE-SI). Candidate SNPs were ranked by PE, and the highest-ranked SNPs were selected to form a 96-SNP panel for each species. The panel size was predefined at 96 single-nucleotide polymorphisms (SNPs) to evaluate whether a compact set of informative markers could achieve parentage assignment performance comparable to that of larger marker panels. Compact panels may offer practical advantages, including the potential to reduce genotyping costs, depending on the assay and platform used. The choice of 96 SNPs also considered the format of targeted genotyping approaches, including KASP assays and custom nanofluidic systems. The selected panels were subsequently evaluated against reference panels and full species-specific datasets.

Step 4: Evaluation of the final panels. The final species-specific panels, each comprising 96 SNPs, were evaluated using four complementary approaches. First, theoretical exclusion power was assessed by calculating cumulative probabilities of exclusion (CPE) in GenAlEx v6.5 [28], using the formula CPE = 1 − ∏(1 − *PEᵢ*), where *PEᵢ* denotes the exclusion probability of the *ith* locus, estimated from allele frequencies in samples pooled across breeds. CPE was calculated under three parentage scenarios: P1, exclusion of a candidate parent when the other parent is known; P2, exclusion of a candidate parent when the other parent is unknown; and P3, exclusion of a candidate parent pair. The minimum number of SNPs required to reach the predefined CPE threshold of 0.9999 in each scenario was determined from the cumulative output generated by GenAlEx. Because the final 96-SNP panels comprise the highest-ranked SNPs by individual PE, the minimum number required to reach this threshold is represented within each panel. The CPE analysis was used to assess the minimum marker requirements for the specified exclusion threshold, rather than to define the final panel size, which was set a priori at 96 SNPs.

The theoretical CPE calculation assumes independence among loci, Hardy–Weinberg equilibrium, and allele frequencies representative of the population in which parentage testing is performed. Because allele frequencies were estimated from samples pooled across breeds, these estimates characterize the combined sample and may not reflect exclusion power within individual breeds. Pooling populations with different allele frequencies may also lead to departures from Hardy–Weinberg proportions in the combined sample. The calculation does not explicitly account for genotyping errors, missing genotypes, null alleles, or relatedness between candidate and true parents. LD pruning and HWE filtering were applied during quality control to reduce potential departures from the assumptions of locus independence and Hardy–Weinberg equilibrium, respectively, but do not guarantee that these assumptions are fully satisfied. Accordingly, CPE estimates represent theoretical exclusion power and should not be interpreted as observed parentage assignment accuracy.

The other evaluation approaches comprised (ii) comparisons of locus-level genetic diversity and non-exclusion metrics with those of random 96-SNP subsets, published reference panels, and the full post-QC species-specific datasets (Section 2.3); (iii) empirical parentage assignment using trios with confirmed pedigree records (Section 2.4); and (iv) assignment performance in simulations under three population-relatedness scenarios (Section 2.4). The empirical trios and simulation datasets comprised animals independent of those used for SNP selection (Section 2.1). Detailed information on the final 96-SNP panels for sheep and goats is provided in Supplementary Table S2.

### 2.3. Comparison of SNP Panels and Statistical Analysis

To evaluate marker informativeness, the optimized 96-SNP panels were compared with two benchmark sets for each species: (i) the full post-QC platform datasets derived from the EMBRAPA Multispecies 65K Illumina Infinium 1 chip (2762 sheep and 1489 goat autosomal SNPs), and (ii) published independent parentage panels (163 sheep SNPs [9] and 195 goat SNPs [17]). All QC filters and the consequent use of the panels are demonstrated in a workflow (Figure 1).

All statistical analyses and graphical visualization were performed in R version 4.5.2 [29] (*readxl*, *tidyverse* [30], *effectsize* [31], and *ggplot2* [32]), using a fixed random seed (123) for reproducibility. Genetic diversity metrics (*H_o_*, *H_e_* and PIC), as well as non-exclusion probabilities (NE-1P, NE-2P, NE-PP, NE-I and NE-SI), HWE deviations, and mean null allele frequencies [F(Null)] were extracted for inter-panel comparisons. Because the 96-SNP panels were selected for informativeness, these comparisons were used to verify the effect of the selection procedure on marker informativeness, and not as a test of parentage assignment performance, which was evaluated separately (Section 2.4).

Because the 96-SNP panel is a curated subset of the full array, the two marker sets are not independent, precluding the use of standard two-sample tests. Therefore, a Monte Carlo randomization test (10,000 iterations of 96 random loci sampled without replacement) was used to test if the 96-SNP panel outperformed random expectations with empirical *p*-values calculated as (b + 1)/(N + 1) [33]. In contrast, comparisons with the independent reference panels, which shared no loci with the selected panels, were performed using two-sided Mann–Whitney *U* tests, reporting effect sizes as absolute rank-biserial correlation (|r|). Per species, all resulting *p*-values were adjusted using the Holm procedure.

### 2.4. Empirical Validation and Simulation Analysis

The SNP-selection, empirical trio-validation, and simulation datasets comprised different individuals, with no overlap among the three sets. Although the same breeds were represented across datasets, different individuals were used for each analysis. As depicted in Figure 1, the performance of the candidate SNP panels was evaluated through a dual empirical validation framework in CERVUS v3.0.7 [27], which assigns parentage based on log-likelihood ratios (LOD scores). Parentage assignment performance was first evaluated using trios with recorded pedigrees (84 ovine trios and 95 caprine trios; samples not used during SNP selection; Section 2.1). Critical LOD and Δ (Delta) score thresholds were established via simulation following Marshall et al. [34], parameterized with 10,000 offspring, a candidate parent sampling proportion of 90%, a typing rate of 90% and a genotyping error rate of 1%. Parentage assignments were categorized at both relaxed (80%) and strict (95%) confidence levels (chosen to assess assignment sensitivity in empirical datasets). Assignment rates, LOD scores, and Mendelian mismatch counts were compared across panel densities (96-SNP panels, reference panels, and full arrays).

Importantly, Mendelian mismatches were defined as genotype incompatibilities between offspring and their assigned parent(s) that could not be explained by Mendelian inheritance. Mismatch counts were obtained from the CERVUS v3.0.7 output. Loci lacking sufficient genotype information for comparison were not included among the loci compared, as reflected in the corresponding output fields. Observed mismatches were not independently verified by re-genotyping and were therefore not considered confirmed genotyping errors. Such incompatibilities may reflect genotyping errors, null alleles, or pedigree discrepancies. The 1.0% genotyping error rate used in the simulation analyses was an assumed model parameter, not an empirical estimate derived from these data.

Subsequently, simulation analyses were performed to evaluate assignment success under realistic breeding conditions without prior trio specifications. Simulations used independent validation cohorts for each species (*n* = 357 for sheep and *n* = 117 for goats; samples not used during SNP selection; Section 2.1). Three scenarios were defined in CERVUS by setting the Relatedness of true parents parameter to 0.00, 0.08, and 0.12, and the Rate of inbreeding in population parameter to 0.00, 0.12, and 0.25, respectively. These scenarios represented outbred, moderately inbred, and highly inbred populations, respectively. Candidate-parent relatedness was additionally evaluated using the Relatives parameter, with proportions of related candidate parents set to 0, 0.2, and 0.5. For each combination of scenario, candidate-parent relatedness, and candidate sampling proportion, CERVUS simulated 10,000 offspring under two candidate-parent sampling rates (0.90 and 0.95), applied equally to candidate sires and dams. All simulations assumed a locus typing rate of 99% and a genotyping error rate of 1.0%. Assignment success, defined as the percentage of true parents correctly identified, was evaluated separately for sires and dams at relaxed (95%) and strict (99%) confidence levels (chosen to ensure high stringency against false positives under complex relatedness scenarios). The optimized 96-SNP panels were compared with the reference panels and full species-specific datasets under identical simulation settings.

The empirical trio validation and simulation analyses used different confidence thresholds to evaluate assignment performance under their respective validation settings; therefore, assignment rates should be interpreted within each analysis rather than directly compared across analyses.

## 3. Results

### 3.1. Informative Performance of the Proposed 96-SNP Panels

The optimized 96-SNP panel developed here for locally adapted Brazilian sheep breeds reached a cumulative exclusion probability (CPE) of 0.9999 under all three evaluated parentage scenarios. Reaching this threshold required 45 markers under P1 (excluding a candidate parent when the other parent is known), 70 markers under P2 (excluding a candidate parent when the other parent is unknown), and 28 markers under P3 (excluding a parental pair). By comparison, the 163-SNP reference panel developed by Heaton et al. [9] required 48, 83, and 30 SNPs to achieve C*PE* = 0.9999 under P1, P2 and P3, respectively. When evaluating the full species-specific dataset (2762 SNPs post-QC), the same threshold was reached with 49 (P1), 88 (P2) and 30 (P3) markers (Table 1).

**Figure 2:**
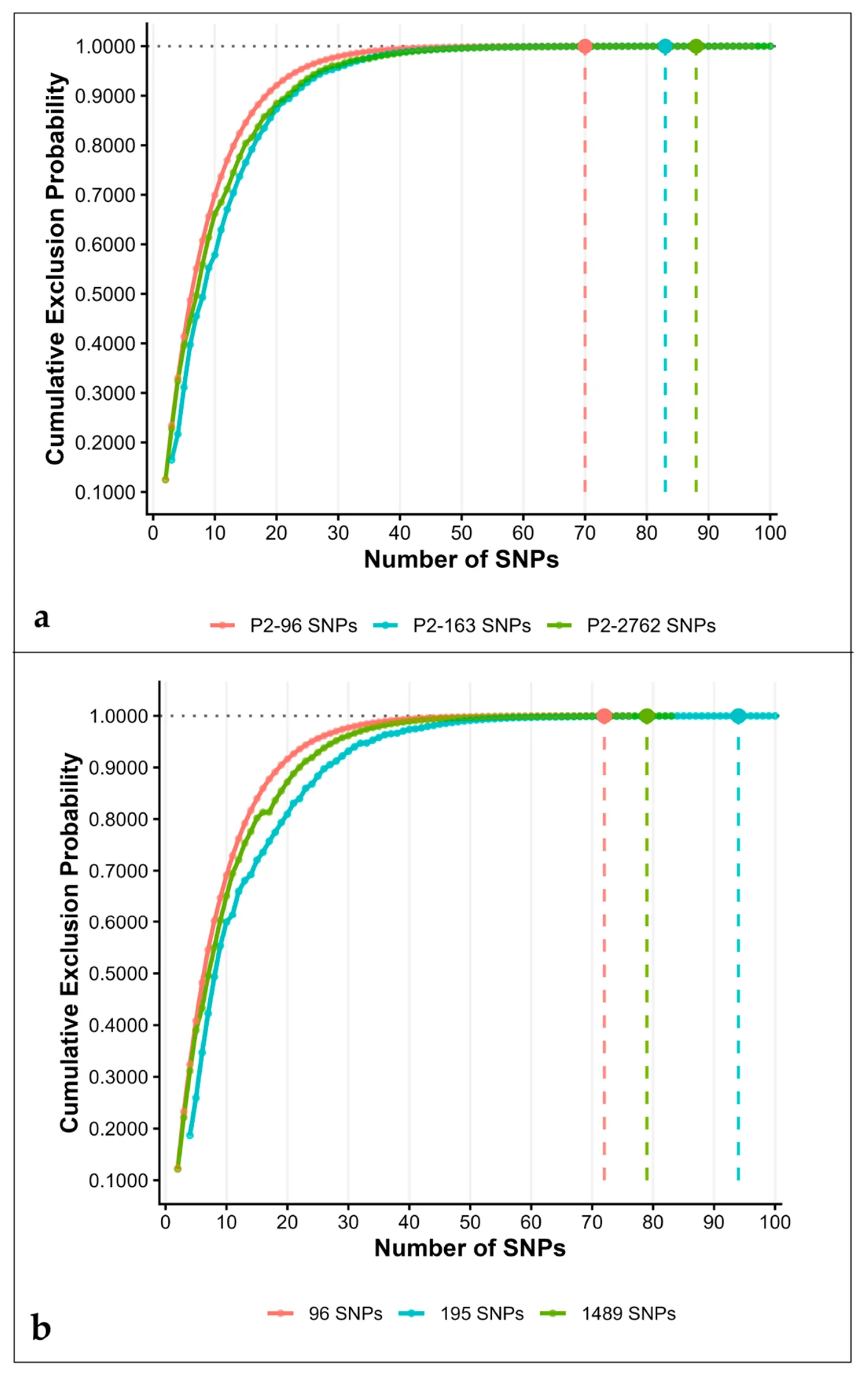
Cumulative probability of exclusion (CPE) under the P2 parentage-exclusion scenario, in which the second parent’s genotype is unknown, across varying marker counts for the three SNP panels evaluated in sheep (**a**) and goats (**b**).

Direct comparison under the P2 parentage-exclusion scenario, in which the second parent’s genotype is unknown, highlighted the informativeness of the optimized panel (Figure 2a). The 96-SNP panel achieved CPE = 0.9999 using 70 SNPs, compared to 83 SNPs for the 163-SNP reference panel and 88 SNPs for the 2762-SNP dataset. Thus, the optimized panel reached the threshold using 13 and 18 fewer markers than the 163-SNP and full datasets, respectively, demonstrating that it concentrated highly informative markers into a substantially smaller marker set.

Similarly, in goats, the optimized 96-SNP panel achieved CPE = 0.9999 with 45 SNPs under P1, 72 under P2, and 29 under P3. When tested in the same Brazilian goat populations, the 195-SNP reference panel [17] required 52, 94, and 33 SNPs to reach the threshold under P1, P2, and P3, respectively. In the full species-specific dataset (1489 SNPs post-QC), CPE = 0.9999 was attained with 48 (P1), 79 (P2) and 31 (P3) markers. Under the P2 scenario (Figure 2b), the 96-SNP panel reached the threshold with 72 SNPs, requiring seven fewer markers than the full dataset (79 SNPs) and 22 fewer than the 195-SNP reference panel (94 SNPs). The lower efficiency of the 195-SNP panel likely reflects divergent genetic backgrounds between the American and European breeds used for its initial development and the locally adapted Brazilian goat breeds evaluated here.

Across both species, the optimized 96-SNP panel demonstrated the highest utilization efficiency among all evaluated sets. Under the P2 scenario, it utilized 72.9% (sheep) and 75.0% (goats) of its total markers to hit CPE = 0.9999 compared to 50.9% and 48.2% for the commercial reference panels, and only 3.2% and 5.3% for the full-density datasets (Table 1). Although the predefined panels contained 96 SNPs, the CPE threshold was reached using 70 markers in sheep and 72 in goats under the P2 scenario. This high efficiency was accompanied by broad genomic coverage: the 96 selected SNPs were distributed across all 26 ovine autosomes (sheep reference genome ARS-UI_Ramb_v3.0; NCBI RefSeq accession GCF_016772045.2) and all 29 caprine autosomes (goat reference genome ARS1.2; NCBI RefSeq accession GCA_001704415.2). Genomic coordinates, chromosome distribution, and markers identifiers for both panels are provided in Supplementary Table S2.

### 3.2. Comparative Genetic Informativeness of Parentage SNP Panels

**Figure 3:**
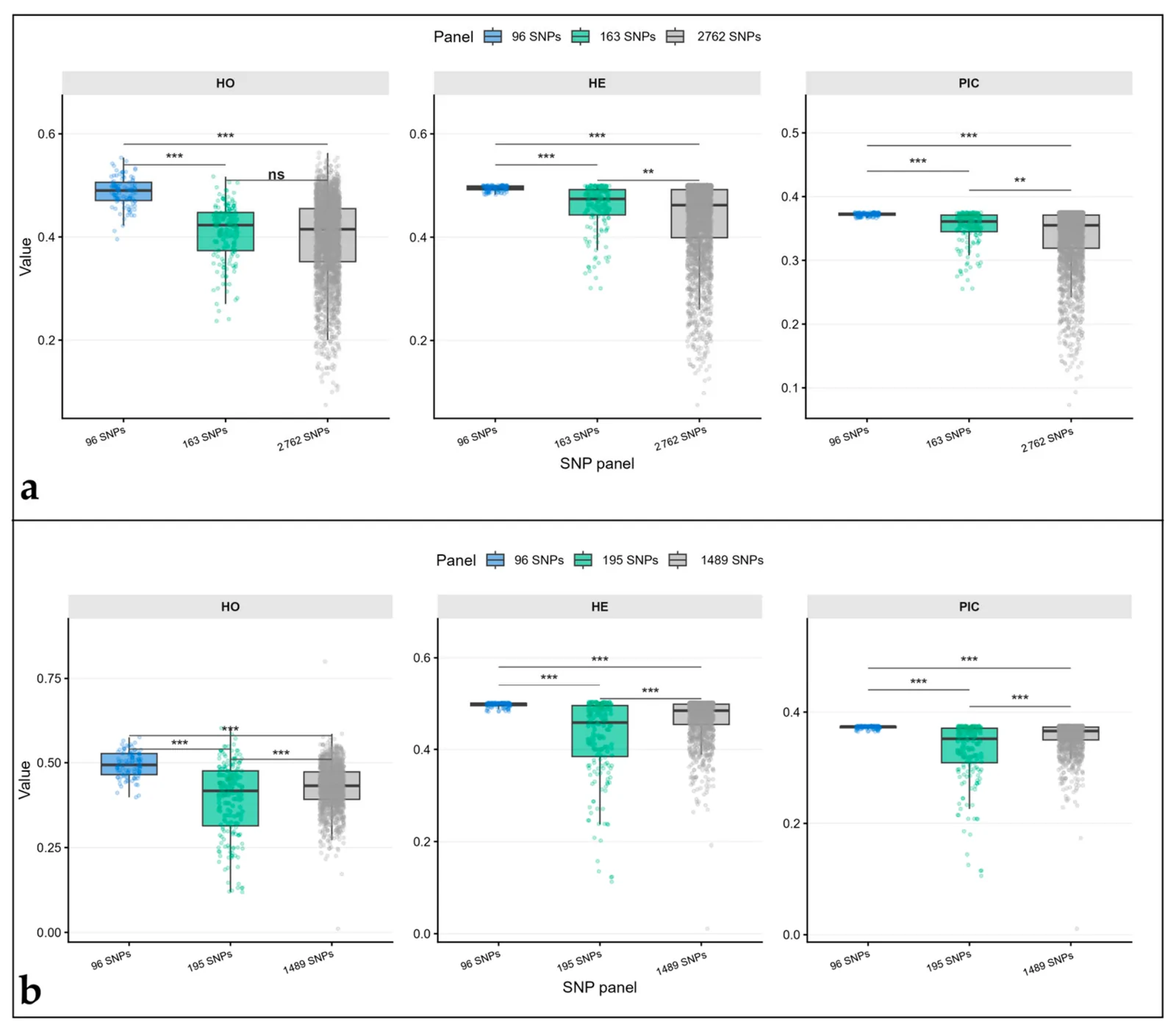
Distribution of locus-level observed heterozygosity (*H_o_*), expected heterozygosity (*H_e_*), and polymorphic information content (PIC) across SNP panels in (**a**) sheep (96, 163, and 2762 SNPs) and (**b**) goats (96, 195, and 1489 SNPs). Each point represents one SNP. In both species, the 96-SNP panel was compared with the respective full array using a Monte Carlo randomization test (10,000 iterations), whereas comparisons involving the independent panels (163 SNPs in sheep and 195 SNPs in goats) were performed using two-sided Mann–Whitney *U* tests. All *p*-values were jointly adjusted for multiple comparisons within each species (*n* = 9 tests per species) using the Holm procedure (*** *p* < 0.001; ** *p* < 0.01; ns, not significant). Full test statistics and effect sizes are presented in Table 2.

Genetic diversity metrics varied among the SNP panels in both species (Table 2; Figure 3). For comparisons between the optimized 96-SNP panels and the full species-specific datasets, the observed mean diversity metrics were evaluated against empirical null distributions of means generated from random 96-locus subsets. Comparisons involving the published reference panels were based on observed median values and assessed using two-sided Mann–Whitney *U* tests.

As expected from the selection criteria (high MAF and PE), the optimized 96-SNP panel showed higher locus-level observed heterozygosity (*H_o_*), expected heterozygosity (*H_e_*), and polymorphic information content (PIC) than expected under random marker selection (Monte Carlo randomization test, 10,000 iterations; Holm-adjusted *p* < 0.0001 for all three metrics in both species). Relative to the published reference panels (163 SNPs for sheep and 195 SNPs for goats), the optimized panel also showed significantly higher values, with large effect sizes (two-sided Mann–Whitney *U* tests; Holm-adjusted *p* < 0.0001; absolute rank-biserial correlations, |r| = 0.83, 0.65, and 0.64 in sheep, and |r| = 0.61, 0.61, and 0.69 in goats, for *H_o_*, *H_e_*, and PIC, respectively).

A different pattern emerged when the published reference panels were compared with the full species-specific datasets. In sheep, *H_o_* did not differ between the 163-SNP and 2762-SNP sets (Holm-adjusted *p* = 0.302; |r| = 0.05), and although *H_e_* and PIC differed significantly (Holm-adjusted *p* = 0.016 for both), the effect sizes were negligible (|r| = 0.12 and 0.13), indicating minimal practical difference. In goats, all three metrics differed significantly between the 195-SNP and 1489-SNP sets (Holm-adjusted *p* < 0.001 for *H_o_* and *p* < 0.0001 for *H_e_* and PIC), with the full dataset showing higher values. However, effect sizes were small to moderate (|r| = 0.16, 0.25, and 0.29 for *H_o_*, *H_e_*, and PIC, respectively), indicating only a modest reduction in locus-level informativeness of the reference 195-SNP panel relative to the full array. Importantly, these locus-level metrics describe marker informativeness and are not, on their own, a measure of parentage assignment accuracy, which is evaluated in Section 3.3.

To characterize locus-level performance, non-exclusion probabilities (NE-1P, NE-2P, NE-PP, NE-I, and NE-SI) were summarized as arithmetic means across all loci within each panel (Table 3). These means were calculated by summing the locus-specific estimates and dividing by the number of loci in each panel. Therefore, the reported values describe the average locus-specific non-exclusion probabilities and should not be interpreted as cumulative probabilities for the complete panel. The 96-SNP panels showed low mean locus-specific non-exclusion probabilities across the evaluated parentage scenarios, although values varied among probability classes and between species. The larger reference panels generally showed lower mean locus-specific non-exclusion probabilities for several metrics, particularly in sheep, whereas the differences among goat panels were less uniform.

No loci in the optimized 96-SNP panels showed significant deviations from Hardy–Weinberg equilibrium (HWE), corresponding to 0/96 loci (0.00%) in both species. In comparison, HWE deviations were observed in 61/163 loci (37.42%) and 275/2762 loci (9.96%) in the sheep reference panel and full dataset, respectively, and in 12/195 loci (6.15%) and 30/1489 loci (2.02%) in the corresponding goat panels. The absence of HWE deviations in the optimized panels is consistent with the HWE-based filtering applied during marker selection. Mean estimated null allele frequencies (F(Null)) were 0.007 in sheep and 0.010 in goats for the optimized panels, compared with 0.057 and 0.047 in the respective reference panels and 0.044 in both full datasets.

### 3.3. Evaluation of the Performance of the Developed Panels

Empirical evaluation using trios with recorded pedigrees demonstrated high parentage assignment success across all SNP panel densities in both species, with assignment rates consistently exceeding 95% at the 95% confidence level (Table 4). In sheep, correct parentage assignment was achieved in 81 out of 84 trios (96.43%) using both the optimized 96-SNP panel and the full 2762-SNP dataset, compared to 80 out of 84 trios (95.24%) for the 163-SNP reference panel (3 unresolved or misassigned trios in the 96-SNP panel vs. 4 in the 163-SNP panel). Because the exact same 84 trios were evaluated across all three panels, pairwise McNemar tests for dependent proportions were conducted to assess differences in assignment performance. No statistically significant differences in assignment success were observed between the optimized 96-SNP panel and the 163-SNP reference panel (*p* = 1.000) or the full dataset (*p* = 1.000).

Similarly, in goats, the optimized 96-SNP panel correctly assigned 91 out of 95 trios (95.80%), matching the performance of the full 1489-SNP dataset (91/95, 95.80%), whereas the 195-SNP reference panel assigned 92 out of 95 trios (96.84%) (4 unresolved trios in the 96-SNP panel vs. 3 in the 195-SNP panel). Pairwise McNemar tests confirmed no significant differences in assignment performance among goat panels (*p* = 1.000) for all comparisons.

Full species-specific datasets yielded higher mean trio LOD scores (16.8 ± 0.18 in sheep and 16.2 ± 0.06 in goats) than the reduced panels. However, this increased statistical likelihood support did not yield a statistically significant increase in empirical assignment accuracy. Moreover, the broader range of Mendelian mismatches observed in the full dataset (up to 7 in sheep and 8 in goats) corresponded to a low observed mismatch rate (<1%) relative to total marker count. A small number of observed SNP mismatches may reflect technical or biological factors and should not be interpreted as confirmed genotyping errors without independent verification [35,36].

Simulation analyses varying candidate sampling proportions, candidate-parent relatedness, and population inbreeding scenarios further evaluated the performance of the optimized 96-SNP panels (Table 5). Across all three panel densities, sire and dam assignment rates remained high at both the relaxed (95%) and strict (99%) confidence levels. Assignment rates primarily reflected candidate sampling proportions, averaging approximately 90% and 95% at sampling rates of 0.90 and 0.95, respectively. Assignment success remained broadly stable across the evaluated proportions of related candidate parents and the three population inbreeding scenarios (*F*_POP_ = 0.00, 0.12, and 0.25). Overall, the optimized 96-SNP panels showed assignment performance comparable to that of the reference panels and full species-specific datasets.Table 5**.** Simulated parentage assignment performance across three SNP panel densities in Brazilian sheep and goats under varying candidate sampling rates, candidate-parent relatedness, and population inbreeding scenarios.

## 4. Discussion

### 4.1. Marker Selection, Genetic Informativeness, and Operational Redundancy

Microsatellite markers (STRs) have long been used for parentage testing in small ruminants [5,37,38,39,40]. However, single-nucleotide polymorphism (SNP)-based approaches offer advantages in cross-laboratory reproducibility, uniform genomic distribution, automated high-throughput scoring, and overall scalability [41,42]. Despite these advantages, locally adapted Brazilian sheep and goat breeds have remained comparatively underrepresented in previous panel development initiatives [9,11,12,13,14,15,16,17]. The present study directly addressed this gap by designing and empirically validating species-specific, optimized 96-SNP panels tailored for parentage assignment in Brazilian small ruminants using genotype data generated with the EMBRAPA Multispecies 65K Illumina Infinium 1 chip.

Comparative analyses showed that the selected 96-SNP sets exhibited significantly higher locus-level observed heterozygosity (*H_o_*), expected heterozygosity (*H_e_*), and polymorphic information content (PIC) than random 96-locus subsets or published reference panels (163 SNPs in sheep; 195 SNPs in goats) [9,17]. This result is consistent with the marker-selection criterion of MAF ≥ 0.30 across all breeds included in marker selection, which was intended to mitigate potential ascertainment bias associated with the underrepresentation of Brazilian breeds in the original reference-panel validation cohorts [9,17]. Crucially, however, locus-level informativeness and parentage assignment performance are related but distinct properties; higher *H_o_*, *H_e_*, and PIC do not, by themselves, demonstrate superior parentage accuracy. When assignment performance was evaluated independently, using empirical trios with confirmed pedigrees and in silico simulations on animals excluded from marker selection, the 96-SNP panels performed comparably to both reference panels and full species-specific datasets (approximately 95–97%). This demonstrates that the selected 96-SNP panels achieved high observed assignment success with fewer markers than the full species-specific datasets, with no statistically significant differences detected in the paired comparisons. However, the absence of statistically significant differences should not be interpreted as evidence of statistical equivalence.

The use of a MAF threshold of ≥ 0.30 across all breeds included in marker selection prioritized highly informative SNPs but imposed a stringent constraint on candidate marker availability. Although relatively balanced allele frequencies can increase the exclusion power of individual loci, this criterion may exclude markers that are informative in particular breeds but less polymorphic in others. This trade-off is relevant when designing compact panels for genetically diverse populations, as allele frequencies and resulting parentage assignment performance may vary among breeds. Accordingly, the selected panels represent a compromise between locus-level informativeness and cross-breed applicability, rather than evidence of equivalent performance across all populations. Further evaluation using larger and more balanced samples from locally adapted Brazilian breeds would help determine the extent to which this selection strategy generalizes across populations.

Under all evaluated parentage scenarios, the optimized 96-SNP panels reached the predefined cumulative exclusion probability threshold (CPE ≥ 0.9999) with fewer than 96 markers. Nevertheless, retaining the full set of 96 SNPs serves an essential operational purpose rather than a purely statistical one. Routine parentage diagnostic workflows must tolerate low call rates, occasional assay failures, locus-specific dropouts, and subtle allele frequency shifts across unmodeled herds or crossbred populations. Designing a panel with additional markers beyond the theoretical minimum introduces necessary redundancy and operational flexibility, aligning with established design principles for international livestock parentage standards [10].

### 4.2. Empirical Parentage Assignment Performance Across Marker Densities

Despite the higher marker density and lower individual non-exclusion probabilities (NE-P) of the full species-specific datasets (2762 SNPs in sheep and 1489 SNPs in goats), these larger datasets did not improve empirical parentage assignment success relative to the optimized 96-SNP panels. In sheep, the 96-SNP panel achieved an assignment success rate of 96.43%, compared with 95.24% for the 163-SNP reference panel and 96.43% for the full dataset. In goats, the 96-SNP panel achieved 95.80% assignment success, matching the full dataset but falling slightly below the 195-SNP reference panel (96.84%). Thus, the results do not demonstrate universal superiority of the optimized panels; rather, they indicate that the 96-SNP panels achieved high empirical assignment success with substantially fewer markers under the conditions evaluated. A similar pattern has been reported in bighorn sheep and Mexican gray wolves [43].

The extent to which additional loci improve assignment depends on factors beyond marker number, including marker informativeness, candidate-parent sampling, and relatedness among candidates. This interpretation is broadly consistent with the simulation results across the three scenarios of parental relatedness and population inbreeding (Scenarios 1–3). Although the full datasets yielded higher mean LOD scores, they also produced higher absolute numbers of Mendelian mismatches, consistent with the larger number of loci evaluated. These mismatches should be interpreted as observed genotype incompatibilities rather than confirmed genotyping errors, because no independent re-genotyping was performed. Finally, the simulation results indicated that candidate-parent sampling completeness influenced assignment success, with changes in assignment performance when the candidate-parent sampling proportion increased from 0.90 to 0.95, consistent with previous livestock parentage evaluations [44].

### 4.3. Practical Implementation and Economic Accessibility

From an applied perspective, the objective of this study was not to supersede high-density genomic arrays, but to establish a basis for evaluating the potential use of these panels in routine parentage testing when full genome-wide profiling is not feasible. The selected 96-SNP panels may be suitable for further evaluation in targeted low-density genotyping platforms, such as KASP (Kompetitive Allele Specific PCR), multiplex microfluidics or targeted sequencing-by-binding assays. This accessibility is particularly relevant to smallholder production systems in South America, which house the majority of locally adapted small ruminant breeds but face strict economic constraints regarding high-density array adoption [21,22,37,38].

A distinct advantage of the proposed 96-SNP panels lies in their origin: all selected markers reside on the EMBRAPA Multispecies 65K Illumina Infinium 1 chip. Unlike the previously developed 163- and 195-SNP panels, which require separate species-specific OvineSNP50 and CaprineSNP50 array genotyping, these 96-SNP panels can be extracted directly from a single multispecies array run. As previously suggested by Rodrigues et al. [26], this offers potential logistical flexibility for diagnostic laboratories and conservation breeding programs, particularly when the multispecies array has already been used for genotyping. In the short term, restricting downstream analyses to these 96 loci may reduce computational requirements while retaining the parentage assignment performance observed in this study.

Nevertheless, some practical and economic caveats must be considered regarding real-world translation. Specifically, evaluating subsets of SNPs extracted from an existing high-density genotyping array demonstrates analytical feasibility and genetic retention, but it does not, by itself, directly capture the manufacturing costs, assay development overhead, or economies of scale associated with a dedicated commercial low-cost assay. Future validation using custom assay formats will be required to confirm their real-world economic advantages.

### 4.4. Study Limitations and Future Directions

A limitation of the empirical caprine validation concerns the unequal representation of breeds, with Saanen accounting for 70 of the 95 evaluated trios (73.7%). This imbalance is consistent with the practical challenges of obtaining sufficient numbers of empirical parentage trios from locally adapted Brazilian goat breeds, which are often maintained in small herds by smallholder farmers or within conservation programs. The limited number of animals available in these populations can constrain the identification and sampling of complete parentage trios, whereas the commercial importance of Saanen as a dairy breed facilitates its representation in larger validation cohorts. Consequently, the empirical results provide evidence of panel performance in the sampled Saanen and locally adapted Brazilian goat populations, but do not establish equivalent validation across all Brazilian goat breeds. In addition, the independent simulation analysis supported the panel’s performance. However, further studies involving larger empirical trio cohorts from locally adapted Brazilian goat breeds are recommended.

Other limitations of this study warrant consideration. First, panel performance was evaluated in purebred populations; thus, diagnostic sensitivity in crossbred or highly admixed animals remains to be formally established. Second, while the 96 loci were validated in silico and empirically via array extraction, downstream translation into a standalone, low-cost physical assay (e.g., KASP or microfluidic panel) was beyond the scope of the present study and requires platform-specific assay optimization. Consequently, analyzing a subset of SNPs from an existing high-density array demonstrates analytical feasibility rather than the direct cost-effectiveness of a dedicated low-cost assay, which should be addressed in future commercial validation studies.

## 5. Conclusions

This study provides empirical evidence that species-specific, optimized 96-SNP panels deliver high parentage assignment accuracy in the evaluated Brazilian sheep and goat populations (96.43% and 95.80%, respectively), performing comparably to larger reference sets and full genome-wide arrays despite a substantial reduction in marker number. These findings support the potential use of the panels for genetic management, pedigree verification, and conservation in the locally adapted breeds evaluated in this study.

Because all selected markers are present on the EMBRAPA Multispecies 65K Illumina Infinium 1 chip, they can be extracted retrospectively from existing datasets generated with this array, potentially enabling parentage verification without additional genotyping for those samples. This approach may be particularly useful for smallholder production systems and conservation nucleus herds, although its broader practical and economic advantages require further assessment.

While this study provides an empirical proof of concept within the evaluated populations and datasets, validation using dedicated low-density diagnostic platforms (e.g., KASP or microfluidic assays), as well as testing across additional Brazilian breeds and admixed or crossbred populations, remain necessary before broader applicability can be established. Overall, these findings provide a basis for further validation of SNP-based parentage testing in Brazilian small-ruminant breeding and conservation programs.

## Figures and Tables

**Table 1:** Minimum number of SNPs required to reach a cumulative probability of exclusion (CPE) of 0.9999 under the P1, P2, and P3 parentage-exclusion scenarios, alongside panel utilization efficiency based on P2, for the three SNP panels evaluated in sheep and goats.

Species	Panel	P1 ^1^	P2 ^2^	P3 ^3^	Marker Utilization Efficiency (P2)
Sheep	96-SNP (Optimized)	45	70	28	72.90%
	163-SNP (Reference) [9]	48	83	30	50.90%
	2762-SNP (Full post-QC)	49	88	30	3.20%
Goat	96-SNP (Optimized)	45	72	29	75.00%
	195-SNP (Reference) [17]	52	94	33	48.20%
	1489-SNP (Full post-QC)	48	79	31	5.30%

^1^ P1: probability of excluding a candidate parent when the other parent’s genotype is known. ^2^ P2: probability of excluding a candidate parent when the other parent’s genotype is unknown. ^3^ P3: probability of excluding a candidate parental pair. Percentage of panel used and additional SNPs were calculated based on P2, the most stringent scenario and therefore the effective minimum panel size required for reliable parentage assignment.

**Table 2:** Statistical comparison of observed heterozygosity (*H_o_*) expected heterozygosity (*H_e_*), and polymorphic information content (PIC) among the SNP panels for sheep and goats.

Species	Comparison	Metric	Value A ^1^	Value B ^2^	Statistic	Test	*p* (Raw)	*p* (Holm)	Effect Size (*r*)
Sheep	96 vs. 2762 SNPs ^1^	*Ho*	0.487	0.398	Mean	Monte Carlo randomization	<0.0001	<0.0001	—
	96 vs. 2762 SNPs ^1^	*He*	0.494	0.434	Mean	Monte Carlo randomization	<0.0001	<0.0001	—
	96 vs. 2762 SNPs ^1^	PIC	0.372	0.337	Mean	Monte Carlo randomization	<0.0001	<0.0001	—
	96 vs. 163 SNPs ^2^	*Ho*	0.490	0.423	Median	Mann–Whitney *U* (*W* = 14,332)	<0.0001	<0.0001	0.830
	96 vs. 163 SNPs ^2^	*He*	0.496	0.474	Median	Mann–Whitney *U* (*W* = 12,898)	<0.0001	<0.0001	0.650
	96 vs. 163 SNPs ^2^	PIC	0.372	0.361	Median	Mann–Whitney *U* (*W* = 12,824)	<0.0001	<0.0001	0.640
	163 vs. 2762 SNPs ^2^	*Ho*	0.423	0.415	Median	Mann–Whitney *U* (*W* = 235,909)	0.302	0.302	0.050
	163 vs. 2762 SNPs ^2^	*He*	0.474	0.462	Median	Mann–Whitney *U* (*W* = 252,842)	0.008	0.016	0.120
	163 vs. 2762 SNPs ^2^	PIC	0.361	0.355	Median	Mann–Whitney *U* (*W* = 254,228)	0.005	0.016	0.130
Goats	96 vs. 1489 SNPs ^1^	*Ho*	0.494	0.429	Mean	Monte Carlo randomization	<0.0001	<0.0001	—
	96 vs. 1489 SNPs ^1^	*He*	0.496	0.468	Mean	Monte Carlo randomization	<0.0001	<0.0001	—
	96 vs. 1489 SNPs ^1^	PIC	0.373	0.357	Mean	Monte Carlo randomization	<0.0001	<0.0001	—
	96 vs. 195 SNPs ^2^	*Ho*	0.494	0.417	Median	Mann–Whitney *U* (*W* = 15,080)	<0.0001	<0.0001	0.611
	96 vs. 195 SNPs ^2^	*He*	0.499	0.458	Median	Mann–Whitney *U* (*W* = 15,090)	<0.0001	<0.0001	0.612
	96 vs. 195 SNPs ^2^	PIC	0.374	0.352	Median	Mann–Whitney *U* (*W* = 15,834)	<0.0001	<0.0001	0.692
	195 vs. 1489 SNPs ^2^	*Ho*	0.417	0.432	Median	Mann–Whitney *U* (*W* = 122,106)	<0.001	<0.001	0.159
	195 vs. 1489 SNPs ^2^	*He*	0.458	0.484	Median	Mann–Whitney *U* (*W* = 109,110)	<0.0001	<0.0001	0.248
	195 vs. 1489 SNPs ^2^	PIC	0.352	0.366	Median	Mann–Whitney *U* (*W* = 102,535)	<0.0001	<0.0001	0.294

^1^ For comparisons marked with ^1^ (96 vs. full species-specific dataset), Value A is the observed mean of the 96-SNP panel, whereas Value B is the mean of an empirical null distribution generated from 10,000 random 96-locus subsets sampled without replacement from the full species-specific dataset (2762 SNPs in sheep; 1489 SNPs in goats). This randomization test was used to determine whether the locus-level genetic diversity of the optimized 96-SNP panel exceeded that expected under random marker selection. ^2^ For comparisons marked with ^2^ (e.g., 96 vs. reference panel or reference panel vs. full species-specific dataset), Value A and Value B correspond to the observed median values of the first and second panels listed in the Comparison column, respectively. These comparisons were performed using two-sided Mann–Whitney *U* tests. Effect sizes are reported as absolute rank-biserial correlations (|r|). For each species, the nine *p*-values were adjusted separately for multiple comparisons using the Holm procedure.

**Table 3:** Mean locus-specific non-exclusion probabilities, number of SNPs deviating from Hardy–Weinberg equilibrium (HWE) and mean estimated null allele frequencies across the evaluated SNP panels in sheep and goats.

Non-Exclusion Probability	Sheep			Goats		
	96 SNPs	163 SNPs	2762 SNPs	96 SNPs	195 SNPs	1489 SNPs
NE-1P ^1^	3.76 × 10^−6^	1.00 × 10^−8^	2.72 × 10^−120^	3.53 × 10^−6^	4.56 × 10^−6^	5.69 × 10^−6^
NE-2P ^2^	2.64 × 10^−6^	2.06 × 10^−11^	3.42 × 10^−219^	2.55 × 10^−6^	5.00 × 10^−13^	8.20 × 10^−11^
NE-PP ^3^	2.20 × 10^−11^	1.02 × 10^−19^	<1.00 × 10^−300^	2.09 × 10^−11^	1.58 × 10^−22^	8.50 × 10^−19^
NE-I ^4^	2.83 × 10^−38^	2.38 × 10^−62^	<1.00 × 10^−300^	2.44 × 10^−38^	1.14 × 10^−69^	<1.00 × 10^−300^
NE-SI ^5^	3.41 × 10^−19^	2.15 × 10^−31^	<1.00 × 10^−300^	3.03 × 10^−19^	5.63 × 10^−35^	7.30 × 10^−30^
SNPs deviating from HWE, *n*/*N* (%)	0 (0.00%)	61 (37.42%)	275 (9.96%)	0 (0.00%)	12 (6.15%)	30 (2.02%)
Mean F(Null)	0.007	0.057	0.044	0.010	0.047	0.044

^1^ NE-1P: Non-exclusion probability for one candidate parent when the other parent is unknown. ^2^ NE-2P: Non-exclusion probability for one candidate parent when the other parent is known. ^3^ NE-PP: Non-exclusion probability for a candidate parental pair. ^4^ NE-I: Probability of identity among unrelated individuals. ^5^ NE-SI: Probability of identity among siblings. The number and proportion of SNPs deviating from HWE are reported as *n*/*N* (%). Mean F(Null) values represent the arithmetic means of locus-specific null allele frequency estimates across each panel. Values represent arithmetic means of the corresponding locus-specific estimates across all SNPs included in each panel and therefore do not represent cumulative panel probabilities. Values < 1.00 × 10^−300^ were below the numerical precision limit used for the calculations.

**Table 4:** Parentage assignment rates, likelihood metrics (LOD and Δ scores), and Mendelian mismatch limits across three SNP panel densities in Brazilian sheep and goats.

Parameters	Sheep (*n* = 84 trios)			Goats (*n* = 95 trios)		
	96 SNPs	163 SNPs	2762 SNPs	96 SNPs	195 SNPs	1489 SNPs
Successful Assignments (*n*)	81	80	81	91	92	91
Unsuccessful/Unresolved (*n*)	3	4	3	4	3	4
Assignment Rate (%) ^1^	96.43%	95.24%	96.43%	95.80%	96.84%	95.80%
McNemar Test (*p*-value vs. 96-SNP)	—	*p* = 1.000	*p* = 1.000	—	*p* = 1.000	*p* = 1.000
Mean Trio LOD Score (±SD)	15.7 ± 0.06	15.6 ± 0.06	16.8 ± 0.18	15.3 ± 0.13	15.3 ± 0.14	16.2 ± 0.06
Mean Trio Δ Score (±SD)	15.6 ± 0.16	15.2 ± 0.21	16.3 ± 0.20	15.0 ± 0.08	15.0 ± 0.19	15.8 ± 0.10
Observed Mismatches per Trio ^2^	0–1	0–2	0–7	0–1	0–1	0–8

^1^ Assignment rate is the proportion of trios correctly assigned at the 95% confidence level, calculated as the number of successful assignments divided by the total number of evaluated trios. SD, standard deviation; Δ (Delta) score, the difference between the LOD score of the most likely candidate and that of the second most likely candidate. ^2^ Mendelian mismatches represent observed genotype incompatibilities reported by CERVUS and were not independently confirmed as genotyping errors.

**Table 5:** Simulated parentage assignment performance across three SNP panel densities in Brazilian sheep and goats under varying candidate sampling rates, candidate-parent relatedness, and population inbreeding scenarios.

Species	Scenario ^1^	Related Candidate Proportion ^2^	Candidate Sampling Rate ^3^	Selected 96-SNP Panels		Reference Panels ^4^		Full Species-Specific Datasets ^5^	
				Sire	Dam	Sire	Dam	Sire	Dam
				(95%/99%)	(95%/99%)	(95%/99%)	(95%/99%)	(95%/99%)	(95%/99%)
Sheep	1	0	0.90	90/90	91/91	90/90	90/90	90/90	91/91
	2	0.2	0.90	91/91	91/91	90/90	90/90	90/90	90/90
	3	0.5	0.90	90/90	91/91	89/89	89/89	90/90	90/90
	1	0	0.95	95/95	96/96	95/95	95/95	94/94	94/94
	2	0.2	0.95	95/95	95/95	95/95	95/95	95/95	95/95
	3	0.5	0.95	95/95	95/95	95/95	95/95	95/95	95/95
Goats	1	0	0.90	90/90	90/90	90/90	90/90	90/90	90/90
	2	0.2	0.90	90/90	90/90	90/90	90/90	90/90	90/90
	3	0.5	0.90	90/90	91/91	90/90	90/90	90/90	90/90
	1	0	0.95	95/95	95/95	95/95	95/95	95/95	95/95
	2	0.2	0.95	95/95	95/95	95/95	95/95	95/95	95/95
	3	0.5	0.95	95/95	95/95	95/95	95/95	95/95	95/95

^1^ Scenario 1: relatedness of true parents = 0.00 and rate of inbreeding in the population = 0.00; Scenario 2: relatedness of true parents = 0.08 and rate of inbreeding in the population = 0.12; Scenario 3: relatedness of true parents = 0.12 and rate of inbreeding in the population = 0.25. ^2^ Proportion of related candidate parents, as specified by the *Relatives* parameter in CERVUS simulations. This parameter is distinct from the relatedness of true parents and the rate of inbreeding in the population. ^3^ Proportion of true candidate sires and dams included in the candidate pool; equal sampling rates were applied to both sexes. ^4^ Reference panels comprised 163 SNPs in sheep and 195 SNPs in goats. ^5^ Full species-specific datasets comprised 2762 SNPs in sheep and 1489 SNPs in goats. Values are percentages of correct assignments across 10,000 simulated offspring, reported at the relaxed (95%) and strict (99%) confidence levels, respectively.

## Data Availability

The data presented in this study are available on request from the corresponding author due to institutional restrictions.

## Supplementary Materials

The following supporting information can be downloaded at: https://www.mdpi.com/article/10.3390/ani16213521/s1, Supplementary Table S1. Summary of the sample sets used in this study. Supplementary Table S2. Information on the SNPs included in the species-specific 96-SNP parentage panels for sheep and goats, including SNP identifiers, allele designations, SNP names, chromosomal locations, and genomic positions.

## References

[1] Rocha A.O., Gloria L.S., Araujo A.C., Wen H., Wilson C.S., Freking B.A., Murphy T.W., Burke J.M., Lewis R.M., Brito L.F. (2025). Genotyping strategies for single-step genomic predictions in a simulated sheep population. Front. Genet..

[2] Luikart G., Biju-Duval M.P., Ertugrul O., Zagdsuren Y., Maudet C., Taberlet P. (1999). Power of 22 microsatellite markers in fluorescent multiplexes for parentage testing in goats (*Capra hircus*). Anim. Genet..

[3] Siwek M., Knol E.F. (2010). Parental reconstruction in rural goat population with microsatellite markers. Ital. J. Anim. Sci..

[4] Ossowski N., Wilkens J., Mendel C., Seichter D., Pohl S., Habermann D., Link E.K. (2022). Analysis of six microsatellite marker sets for parentage testing in four sheep breeds in Germany. Small Rumin. Res..

[5] Karaca O., Yılmaz O., Ata N., Sevim S. (2024). Accuracy in pedigree records in Hair goats: New microsatellite-based paternity test panels. J. Anim. Prod..

[6] International Society for Animal Genetics (ISAG) (2012). Applied Genetics in Sheep and Goats: Workshop Report.

[7] Flanagan S.P., Jones A.G. (2019). The future of parentage analysis: From microsatellites to SNPs and beyond. Mol. Ecol..

[8] Zimmerman S.J., Aldridge C.L., Oyler-McCance S.J. (2020). Empirical comparison of population genetic analyses using microsatellite and SNP data. BMC Genom..

[9] Heaton M.P., Leymaster K.A., Kalbfleisch T.S., Kijas J.W., Clarke S.M., McEwan J., Maddox J.F., Basnayake V., Petrik D.T., Simpson B. (2014). SNPs for parentage testing and traceability in globally diverse breeds of sheep. PLoS ONE.

[10] International Society for Animal Genetics (ISAG) (2013). ISAG Cattle Core and Additional SNP Panel.

[11] Kijas J., van der Werf J., Ferdosi M., Bell A., Gill S., Gore K., Driver F., Maddox J., Henshall J. (2012). SNP-based parentage assignment in sheep: Application in Australian flocks. Proceedings of the 33rd Conference of the International Society for Animal Genetics, Cairns, Australia, 15–20 July 2021.

[12] Clarke S.M., Henry H.M., Dodds K.G., Jowett T.W., Manley T.R., Anderson R.M., McEwan J.C. (2014). A high-throughput single nucleotide polymorphism multiplex assay for parentage assignment in New Zealand sheep. PLoS ONE.

[13] Deniskova T.E., Dotsev A.V., Gladyr’ E.A., Sermyagin A.A., Bagirov V.A., Hompodoeva U.V., Il’in A.N., Brem G., Zinovieva N.A. (2015). Validation of the SNP panel for parentage assignment in local Russian sheep breeds. Agric. Biol..

[14] Tortereau F., Moreno C.R., Tosser-Klopp G., Servin B., Raoul J. (2017). Development of a SNP panel dedicated to parentage assignment in French sheep populations. BMC Genet..

[15] Berry D.P., McHugh N., Wall E., McDermott K., O’Brien A.C. (2019). Low-density genotype panel for both parentage verification and discovery in a multi-breed sheep population. Ir. J. Agric. Food Res..

[16] Calvo J.H., Serrano M., Tortereau F., Sarto P., Iguacel L.P., Jiménez M.A., Folch J., Alabart J.L., Fabre S., Lahoz B. (2021). Development of a SNP parentage assignment panel in some north-eastern Spanish meat sheep breeds. Span. J. Agric. Res..

[17] Talenti A., Palhière I., Tortereau F., Pagnacco G., Stella A., Nicolazzi E.L., Crepaldi P., Tosser-Klopp G., AdaptMap Consortium (2018). Functional SNP panel for parentage assessment in worldwide goat breeds. Genet. Sel. Evol..

[18] Silva R.C., Melo M.N., Oliveira C.F.T., Cardoso J.V., Cevallos L.A.C., Rocha L.L., Arandas J.K.G., Ribeiro M.N. (2025). Ethnozootechnical perspectives on the decline of traditional knowledge about local goat and sheep breeds in the semi-arid region of Paraíba, Brazil. Ruminants.

[19] Tavares da Silva W.S., Silveira R.M.F., Leite J.H.G.M., Bermejo L.A., Krupij A.T., Façanha D.A.E. (2026). Morphological and physiological adaptive profiles of Spanish and Brazilian goat breeds in arid environments. Trop. Anim. Health Prod..

[20] Rodrigues C.S., Faria D.A.d., Azevedo H.C., Silva K.d.M., Facó O., Santos S.A., Braga R.M., Caetano A.R., Moraes J.C.F., Souza C.J.H.d. (2026). Assessment of a Reduced SNP Panel Targeting Prolificacy and Coat Color Genes in Brazilian Sheep Breeds. Animals.

[21] Sousa W.H., Benitez Ojeda M.D., Facó O., Cartaxo F.Q. (2011). Genetic improvement of goats in Brazil: Experiences, challenges and needs. Small Rumin. Res..

[22] Freitas Silveira R.M., Vasconcelos A.M., Silva V.J., Ortiz Vega W.H., Toro-Mujica P., Ferreira J. (2021). Typification, characterization, and differentiation of sheep production systems in the Brazilian semiarid region. NJAS Impact Agric. Life Sci..

[23] Lopes U.V., Pires J.L., Gramacho K.P., Grattapaglia D. (2022). Genome-wide SNP genotyping as a simple and practical tool to accelerate the development of inbred lines in outbred tree species: An example in cacao (*Theobroma cacao* L.). PLoS ONE.

[24] Rodrigues C.S., Faria D.A.d., Ramos A.F., McManus C., Paiva S.R. (2025). Uso de ferramenta genômica no manejo genético das raças conservadas in situ na Embrapa Recursos Genéticos e Biotecnologia.

[25] Guazzeli L.d.M., Araujo A.M.d., Santos S.A., Juliano R.S., Rodrigues C.S., Faria D.A.d., Paiva S.R. (2025). Uso de ferramentas genômicas no manejo do Núcleo de Conservação Animal da Embrapa Pantanal.

[26] Rodrigues C.S., Faria D.A.d., Paiva S.R., McManus C. (2026). Customized SNP Panel for Local Brazilian Sheep Breed Assignment. Genes.

[27] Kalinowski S.T., Taper M.L., Marshall T.C. (2007). Revising how the computer program CERVUS accommodates genotyping error increases success in paternity assignment. Mol. Ecol..

[28] Peakall R., Smouse P.E. (2012). GenAlEx 6.5: Genetic analysis in Excel. Population genetic software for teaching and research—An update. Bioinformatics.

[29] R Core Team (2025). R: A Language and Environment for Statistical Computing.

[30] Wickham H., Averick M., Bryan J., Chang W., McGowan L.D.A., François R., Grolemund G., Hayes A., Henry L., Hester J. (2019). Welcome to the tidyverse. J. Open Source Softw..

[31] Ben-Shachar M.S., Lüdecke D., Makowski D. (2020). effectsize: Estimation of effect size indices and standardized parameters. J. Open Source Softw..

[32] Wickham H. (2016). ggplot2: Elegant Graphics for Data Analysis.

[33] Phipson B., Smyth G.K. (2010). Permutation P-values should never be zero: Calculating exact P-values when permutations are randomly drawn. Stat. Appl. Genet. Mol. Biol..

[34] Marshall T.C., Slate J., Kruuk L.E.B., Pemberton J.M. (1998). Statistical confidence for likelihood-based paternity inference in natural populations. Mol. Ecol..

[35] International Committee for Animal Recording (ICAR) (2017). ICAR Guidelines for Parentage Verification and Parentage Discovery Based on SNP Genotypes.

[36] McClure M.C., McCarthy J., Flynn P., McClure J.C., Dair E., O’Connell D.K., Kearney J.F. (2018). SNP data quality control and highly accurate SNP-based parentage verification and identification. Front. Genet..

[37] Silva E.C., McManus C.M., Guimarães M.P.P., Gouveia A.M., Facó O., Pimentel D.M., Caetano A.R., Paiva S.R. (2014). Validation of a microsatellite panel for parentage testing of locally adapted and commercial goats in Brazil. Genet. Mol. Biol..

[38] Menezes M.P.C., Martinez A.M., Filho E.C.P., Vega-Pla J.L., Delgado J.V., Arandas J.K.G., Rocha L.L., Ribeiro M.N. (2020). Diversity analysis and genetic relationships among local Brazilian goat breeds using SSR markers. Animals.

[39] Souza C.A., Paiva S.R., McManus C.M., Azevedo H.C., Mariante A.S., Grattapaglia D. (2012). Genetic diversity and assessment of 23 microsatellite markers for parentage testing of Santa Inês hair sheep in Brazil. Genet. Mol. Res..

[40] Al-Atiyat R.M. (2015). The power of 28 microsatellite markers for parentage testing in sheep. Electron. J. Biotechnol..

[41] Ajmone-Marsan P., Boettcher P.J., Colli L., Ginja C., Kantanen J., Lenstra J.A. (2023). Genomic Characterization of Animal Genetic Resources: Practical Guide.

[42] Husien H.M., Saleh A.A., Hassanine N.N.A.M., Rashad A.M.A., Sharaby M.A., Mohamed A.Z., Abdelhalim H., Hafez E.E., Essa M.O.A., Adam S.Y. (2024). The evolution and role of molecular tools in measuring diversity and genomic selection in livestock populations. Vet. Sci..

[43] Andrews K.R., Adams J.R., Cassirer E.F., Plowright R.K., Gardner C., Dwire M., Hohenlohe P.A., Waits L.P. (2018). A bioinformatic pipeline for identifying informative SNP panels for parentage assignment from RADseq data. Mol. Ecol. Resour..

[44] Laseca N., Molina A., Marshall T., González I., Poyato-Bonilla J., Valera M. (2026). A breed-specific SNP panel with higher theoretical exclusion power and practical equivalence to international standards for parentage testing in Pura Raza Española horses. Anim. Genet..

